# Computer vision *vs.* spectrofluorometer-assisted detection of common nitro-explosive components with *bola*-type PAH-based chemosensors†

**DOI:** 10.1039/d1ra03108b

**Published:** 2021-07-27

**Authors:** Igor S. Kovalev, Leila K. Sadieva, Olga S. Taniya, Victoria M. Yurk, Artem S. Minin, Sougata Santra, Grigory V. Zyryanov, Valery N. Charushin, Oleg N. Chupakhin, Mikhail V. Tsurkan

**Affiliations:** Ural Federal University named after the first President of Russia B. N. Yeltsin 19 Mira Str., K-2 Yekaterinburg 620002 Russian Federation ekls85@yandex.ru sougatasantra85@gmail.com gvzyryanov@gmail.com; I. Ya. Postovskiy Institute of Organic Synthesis, Ural Division of the Russian Academy of Sciences 22 S. Kovalevskoy Str. Yekaterinburg 620219 Russian Federation; Leibnitz Institute for Polymer Research Dresden 01069 Dresden Germany tsurkan@ipfdd.de; M. N. Mikheev Institute of Metal Physics, Ural Branch of the Russian Academy of Sciences 18 S. Kovalevskoy Str Yekaterinburg 620219 Russian Federation

## Abstract

Computer vision (CV) algorithms are widely utilized in imaging processing for medical and personal electronics applications. In sensorics CV can provide a great potential to quantitate chemosensors' signals. Here we wish to describe a method for the CV-assisted spectrofluorometer-free detection of common nitro-explosive components, *e.g.* 2,4-dinitrotoluene (DNT) and 2,4,6-trinitrotoluene (TNT), by using polyaromatic hydrocarbon (PAH, PAH = 1-pyrenyl or 9-anthracenyl) – based *bola*-type chemosensors. The PAH components of these chemical *bolas* are able to form stable, bright emissive in a visual wavelength region excimers, which allows their use as extended matrices of the RGB colors after imaging and digital processing. In non-polar solvents, the excimers have poor chemosensing properties, while in aqueous solutions, due to the possible micellar formation, these excimers provide “turn-off” fluorescence detection of DNT and TNT in the sub-nanomolar concentrations. A combination of these PAH-based fluorescent chemosensors with the proposed CV-assisted algorithm offers a fast and convenient approach for on-site, real-time, multi-thread analyte detection without the use of fluorometers. Although we focus on the analysis of nitro-explosives, the presented method is a conceptual work describing a general use of CV for quantitative fluorescence detection of various analytes as a simpler alternative to spectrofluorometer-assisted methods.

## Abstract 2

Simplified computer vision-assisted algorithm for the excimer fluorescence "turn-off" detection of nitro-analytes in aqueous media is described.

## Graphical abstract

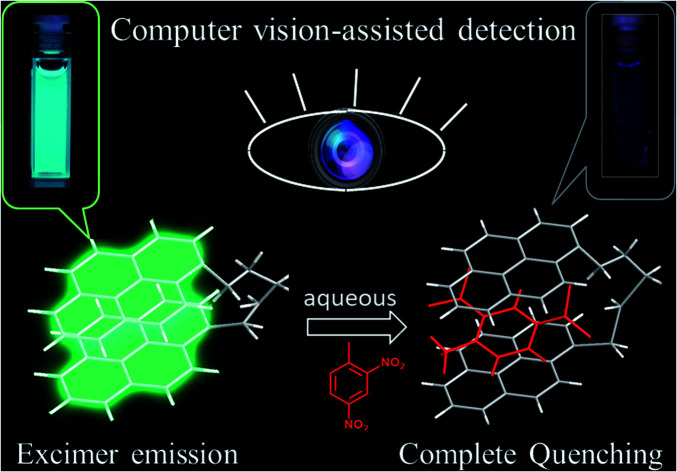

## Introduction

Chemical sensor-based visual detection methods of various analytes (including explosives)^1–4^ are one of the oldest analytical techniques offering vast possibilities for on-site, real-time analysis with a very fast response time. Among visual methods, fluorescence-based ones,^5–8^ such as “turn-off”^9–12^ or, rarely, “turn-on”,^13,14^ have the highest sensitivity with a theoretical single molecule accuracy limit. Together, the high sensitivity and “instant” response time make fluorescence-based techniques more beneficial for practical use compared to other detection methods such as gas chromatography,^15^ high-performance liquid chromatography,^5–8^ (surface-enhanced) Raman spectroscopy,^5–8,16^ infrared and terahertz absorption spectroscopy,^5–8,17^ mass spectrometry,^5–8,18^ photoacoustic spectroscopy^19^ as well as immunoassay^20,21^ and electrochemical techniques.^22^ Fluorescence-based chemosensors/sensory materials are, as a rule, cheap, mobile, and accurate, which is a crucial issue for the development of highly affordable and reliable devices for the detection of various analyses, including nitro-explosives.^23–25^

**Fig. 1:**
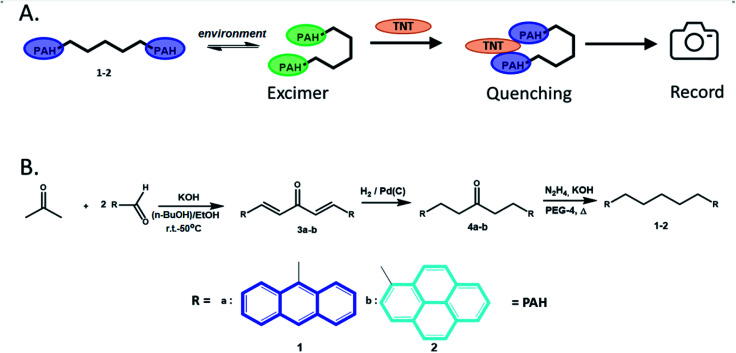
Chemical “*bolas*-type” structures with different end moieties 1 and 2. (A) Concept of the expected nitroaromatic explosive detection by “*bolas*-type” chemosensors; (B) the synthetic scheme for sensors 1–2.

Among many fluorescent chemosensors,^26^ PAH-based (multi)fluorophore ones have attracted much attention due to their remarkable fluorescent properties, such as ability to form excimers^27^ either in a solution^28^ or in a gas phase,^29^ with a strong emission. PAHs are common components for NIR dyes,^30^ OLEDs,^31^ LC displays,^32^ and batteries,^33^ as well as photoresponsive materials^34^ and photovoltaic devices.^30^ Excimer emission of PAH-based fluorophores is a convenient tool for the analysis of water or food samples,^35^ for DNA studies,^36^ as well as for the “turn-off’ detection of various analytes/pollutants,^37^ including (nitro)explosives.^38,39^ In the last case the fluorescence quenching of PAH fluorophores *via* either charge or energy transfer is easily registered by a fluorimeter. Although the intensity of the charge/energy transfer, as well as the absorption and emission maxima in PAH fluorophores are easily tunable, their excimer stabilization is still subjected to extensive studies. And the effective manipulation of the excimer formation requires advanced molecular organization beyond the commonly utilized changing structural environment or pre-concentration/organization in organic/aqueous media. Recently, an interesting molecular design was reported where two PAH fluorophores were linked *via* a hydrocarbon chain^40^ to form chemical *bolas* (Fig. 1A). These chemical *bolas* could bend over, thus, “trapping” such analytes as nitro-explosives resulting in the quenching of PAH-excimer emission due to the formation of a “nitro-explosive-PAH” molecular complex.

Nowadays, terrorism threats are increasing, and explosive detonations in crowded places are challenging to address. TNT and DNT are the main components in the majority of explosive blends.^41^ Therefore, timely detection of these explosives is highly essential for the sake of national security, civilian safety, as well as community protection. It is worthy to mention that, the nitro-explosives and their detonation/decomposition products are highly toxic, and by being left from the explosions or improper disposal of undetonated components these compounds could penetrate easily to the groundwaters and contaminate them. During last decade our researches are focused on the development of new, highly sensitive methods for the nitro-explosives detection by using fluorescence chemosensors. Theoretically, the “turn-off” fluorescence response can be observed by a naked eye,^42^ but as usual, it works only with higher quencher concentrations, and human error is potentially involved. Computer/machine vision is an algorithm when the computer gaining high-level understanding from digital images/videos and could automate and preferably outperform the tasks that the human visual systems can do.^43^ Attempts to digitize the intensity of the colorimetric response *via* computer vision elements^44^ using conventional cameras^45^ have shown to improve sensitivity of the colorimetry-based (nitro)analytes detection compare to the naked eye. However, the precise quantitative analysis still requires the use of spectrophotometers to reveal the exact analyte concentration.

Herein, we are pleased to report our findings on the development of spectrometer-free methods for the nitro-explosives (TNT and DNT) detection by using pyrene or anthracene based *bolas*-type chemosensors possessing with a strong excimer emission in a visual region. We believe that, the application of computer vision algorithms for the quantification of the fluorescent color-intensity alteration could potentially provide the information about the concentration of (nitro)analytes without the use of fluorimeters. To the best of our knowledge, no reports have been made so far on fluorescence-based detection of nitro-explosives *via* computer/machine vision algorithms.

## Results and discussion

Chemosensors 1–2 were prepared according to the previously reported method^40,46^ by using the condensation reaction between the corresponding PAH-based aldehydes and acetone followed by the reduction of the obtained condensation products (Fig. 1B).

Since titration on a spectrofluorometer is carried out by the Single Point method, *i.e.* at one specific predetermined wavelength, it was necessary to use a sensor in which fluorescence quenching occurs either at the monomer wavelength or at the excimer wavelength. Moreover, it is more preferable to quench the excimer emission, because the latter, dynamic in nature, are more sensitive to changes in the environment, and, as a result, gives a more pronounced analytical signal/sensory response. Therefore, our main goals were to select the proper solvent media for providing an enhanced excimer emission for the compounds 1–2 and utilizing these conditions for the detection of nitro-explosives (Fig. 1A).

Previously, it was reported^40^ about the intensive excimer emission of 10^−5^ M solutions of compound 2 in non-polar solvent such as methylcyclohexane. Therefore, as a first step, the photophysical properties of the obtained compounds 1–2 were studied in similar conditions (in cyclohexane), and the fluorescence titration of 2 was carried out in the presence of the most common nitroaromatic explosive (NAE), TNT. Despite the satisfactory linearity of the Stern–Volmer plot, quite feeble response was observed through the excimer fluorescence quenching (see ESI, Fig. S5†). Such weak sensory response can be explained by two factors: (1) according to Zachariasse and Kühnle^40^ (confirmed later by Reiter *et al.*^47^) for 1,5-bis(pyren-1-yl)pentane an optimal “sandwich-like”-configuration of pyrene units cannot be reached, and overlapping is relatively small. As a result, excimeric emission intensity is rather low. It is reflected in one of the smallest *I*_ex_/*I*_m_ values in the Py(CH_2_)_*n*_Py series (where *n* = 2–16, 22) and in strong blue-shift of the maximum of excimeric emission compared to that of bis(pyren-1-yl)propane.^40^ Consequently, quenching of such weak emission show insignificant response; (2) in non-polar media there is no additional driving force for lipophilic NAE to stack with PAHs moieties besides photo-induced electron transfer (PET).

As for anthracene-based compound 1, no excimeric emission was observed in cyclohexane. It can be attributed to shorter fluorescence lifetime and, probably, smaller value for the excimer stabilization energy, compared to pyrene.

Based on the earlier reports,^48,49^ we suggested that the polarity of solvent or hydrophilicity of a medium can be the driving force for the geometry changes in the lipophilic molecules of 1–2. In an aqueous organic or polar environment, these *bolas*-type molecules could act as non-ionic surfactants, which would exhibit an enhanced excimer emission due to the bending of the molecules 1–2 over the pentane linker and the following proximity effect between two PAH molecules, while the monomeric emission of 1–2 would be suppressed. We observed similar effects in cyclic cyclophanes (pillar[6]arenes) in polar solvents media,^50^ in recently reported micellar pyrene-based chemosensors for explosives^51,52^ as well as in chemosensors for anions.^53^ In the micellar chemosensors the local non-polar “friendly” environment inside the body of micelle was the driving force for the less polar molecules, for instance, nitro-aromatic explosives (NAE), to transport from the polar solvent media inside the non-polar cavity of the micellar chemosensor.

**Fig. 2:**
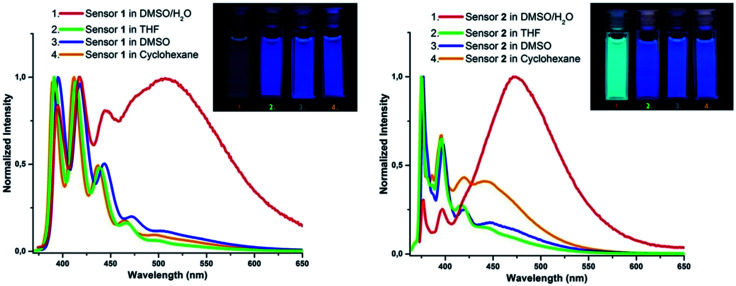
Emission characteristics of the sensors at *λ*_ex_ = 365 nm excitation. Normalized emission spectra of sensor 1 (left) and sensor 2 (right) in the solvents of different polarity. The images are of the corresponding solutions (10^−5^ M) of sensors 1 and 2.

For this purpose, the analysis of the photophysical properties of compounds 1–2 (10^−5^ M) was carried out in different solvent systems of various polarity. The selected solvents were arranged in the order of their increasing polarity: THF, DMSO and DMSO : H_2_O [1 : 1 (v/v)]. The pronounced excimer emission, which suppressed the monomeric emission, was observed only for the DMSO : H_2_O [1 : 1 (v/v)] solution of compound 2 (Fig. 2). Large bathochromic shift compared to excimeric emission maximum of 2 in cyclohexane indicates higher degree of overlap between two pyrene moieties. Among all solvents compound 1 formed excimer only in DMSO : H_2_O [1 : 1 (v/v)].

Based on the analysis of spectral data, the compound 2 was found to be more suitable for further experiments as its emission in aqueous DMSO was mainly represented by only the excimer emission while monomer emission was suppressed (Fig. 2, right). In this case for the both techniques (CV-assisted and spectrofluorometer-assisted ones) the titration would produce comparable results. In case of sensor 1 both type of emissions, such as monomeric and excimeric ones were presented (Fig. 2, left). So, when the fluorescence titration of 1 occurs on the wavelength of excimeric emission a part of the analytical signal corresponding to the quenching of monomeric emission was not taken into account and, accordingly, was subtracted upon the calculation of the Stern–Volmer quenching constant. In case of CV-assisted algorithm the fluorescence quenching of the entire emission spectrum was estimated. As a consequence, we expected a difference in values of quenching constants calculated for the chemosensor 1 by means of spectrofluorometer-assisted and CV-assisted algorithms, and as a consequence, a small difference in those for the sensor 2.

Standard quenching experiments were carried out by using 3 mL cuvettes on the Horiba Fluoromax 4 spectrofluorometer using the excitation wavelength according to the excimer maxima for compounds 1–2. Next, the fluorescence quenching titrations were carried out. To do that, the equal aliquots of the 10^−3^ M (sensor 1) or 10^−4^ M (for sensor 2) TNT solutions were added to the 10^−5^ M (sensor 1) or 10^−6^ M (sensor 2) sensor solutions, and the decrease of the fluorescence of sensors 1–2 was estimated (see ESI, Tables S2–S8†). The fluorescence response of the chemosensors towards the nitro-analyte was calculated using the Stern–Volmer static quenching model according to eqn (1):1
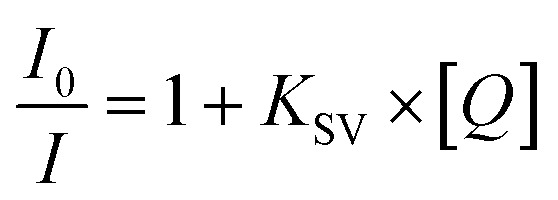
where *I*_0_ is the initial intensity, or rate of fluorescence, without a quencher, *I* is the current intensity, or rate of fluorescence, with a quencher, *K*_SV_ is the Stern–Volmer quenching constant for complex formation, and [*Q*] is the current concentration of the quencher. The quenching constant (*K*_SV_) was calculated as the slope of the graph intensity ((*I*_0_/*I*) − 1) *versus* the concentration of the quencher ([*Q*]).

**Fig. 3:**
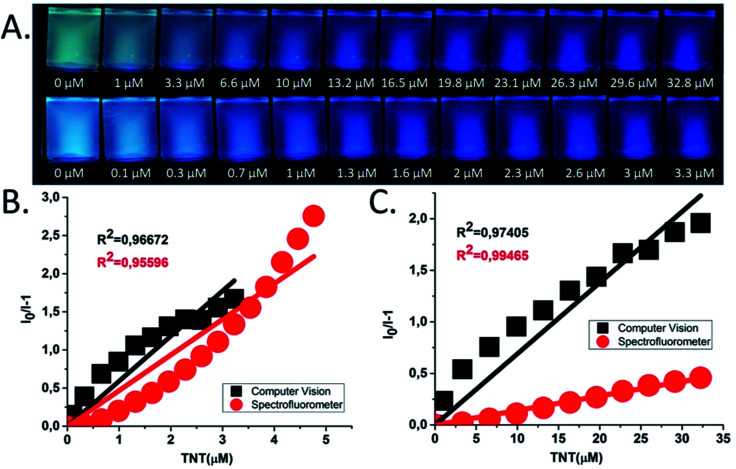
Quenching experiments of chemosensors 1 and 2 in DMSO : H_2_O [1 : 1 (v/v)]: (A) pictures of sensor 1 (upper row) and 2 (lower row) under UV light (*λ* = 365 nm) after stepwise addition of nitro explosive (TNT). (B) Stern–Volmer plots of emission quenching for sensors 2. (C) Stern–Volmer plots of emission quenching for sensors 1.

The Stern–Volmer constant values for TNT were determined to be as high as *K*_SV_ = 14 028 M^−1^ for sensor 1 and *K*_SV_ = 467 274 M^−1^ for sensor 2 for the static quenching model (Fig. 3B and C). Despite the *bolas*-type geometry, sensor 1 exhibited a less intense turn-off fluorescence response (summarized in Table 1) to nitro-explosives compare to sensor 2. Such behavior is attributed to the anthracene's lesser affinity towards nitroaromatics, as it was mentioned previously.^7^ Interestingly, the linear behavior of Stern–Volmer plots was observed in all the cases, which suggests the prevalence of only one quenching mechanism, such as static quenching, at lower concentrations of the quencher.

**Table 1:** Summary of the quenching experiments

#	*C*_TNT_ in the vial of sensor 1, M	*C*_TNT_ in the vial of sensor 2, M	Average intensity on the picture of the sensor 1, a.u.	Average intensity on the picture of the sensor 2, a.u.	*I*_0_/*I* − 1 for the sensor 1	*I*_0_/*I* − 1 for the sensor 2
1	0	0	112.577^a^	113.285^a^	0	0
2	1.00 × 10^−6^	1.00 × 10^−7^	91.294	98.605	0.233	0.149
3	3.33 × 10^−6^	3.33 × 10^−7^	73.111	81.465	0.54	0.391
4	6.64 × 10^−6^	6.64 × 10^−7^	64.139	67.178	0.755	0.686
5	9.95 × 10^−6^	9.95 × 10^−7^	57.623	61.416	0.954	0.845
6	1.32 × 10^−5^	1.32 × 10^−6^	53.362	55.053	1.11	1.058
7	1.65 × 10^−5^	1.65 × 10^−6^	48.927	52.361	1.301	1.164
8	1.98 × 10^−5^	1.98 × 10^−6^	46.199	49.067	1.437	1.309
9	2.31 × 10^−5^	2.31 × 10^−6^	42.263	47.199	1.664	1.4
10	2.63 × 10^−5^	2.63 × 10^−6^	41.735	47.334	1.697	1.393
11	2.96 × 10^−5^	2.96 × 10^−6^	39.227	44.347	1.87	1.554
12	3.28 × 10^−5^	3.28 × 10^−6^	38.055	42.445	1.958	1.669

a *I*_0_, intensity of emission from blank solution.

Next, a spectrofluorometer-free approach for detecting TNT and DNT was explored, aiming to develop a simpler and faster nitro-explosives detection method. The quenching experiments were carried out simultaneously for the solutions of sensors 1 and 2 by using common borosilicate glass vials (10 mL) (Fig. 3A). These blank solutions of sensors 1–2 were placed in a dark chamber under UV lamp at *λ* = 365 nm, and their photos were taken. After that the eleven aliquots of the TNT solution were added stepwise into each vial and photos were taken after adding each aliquot (Fig. 3A). As a final step, all the obtained photos were cropped, and top/bottom centered in order to facilitate further processing on the computer.

The post-processing of the photos was made using Mathcad 15 software (for the detailed algorithm see ESI, page S11†). Twelve pictures of the vials were digitally processed as extended matrices of the RGB colors as follows. Each matrix which is presented one photo was mathematically recalculated according to the formula for the HDTV processing (2) into the matrix of the grayscale picture in which each pixel is represented by the digit from 0 (absolute darkness) to 255 (bright white).

2 *Y*′ = 0.2126*R* + 0.7152*G* + 0.0722*B*

where *Y*′ is the brightness in grayscale; intensities *R* (red), *G* (green), *B* (blue) channels – respectively.

Such recalculation has two advantages: the first, there is no influence of the diffused UV light and monomeric emission on the results because in the eqn (2) blue light is almost neglected by the coefficient 0.0722; the second, green light, and to a lesser degree red light that is present in excimer emission are not weakened in the calculation. In this case, brightness is represented by the intensity of the excimer emission of the solutions. After that, two submatrices *I*, were chosen that cover rectangular zones with brighter intensity on both vials. Data in these matrixes were arithmetically averaged by eqn (3).3
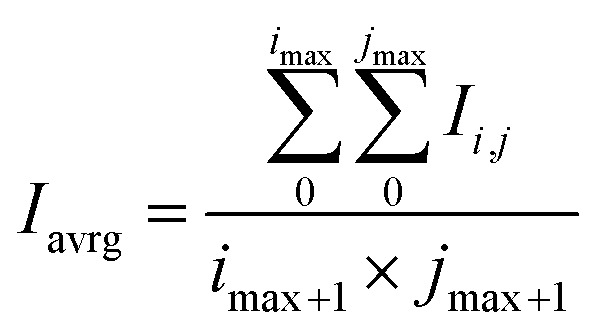
where *I*_avrg_ is the averaged brightness; *i*_max_, *j*_max_ – maximum values of rows and columns of the matrix; *I*_*i*,*j*_ are the values of the *i*-th and *j*-th elements of the matrix *I*.

The data of the average intensity were converted to the Stern–Volmer eqn (1) view meaning *I*_0_/*I* − 1 and all results were summarized in Table 1.

Stern–Volmer constants *K*_SV_ were calculated as coefficients of the linear regression utilizing the least square method for the special case of the function *y* = *b* × *x* according to the formula (4).4
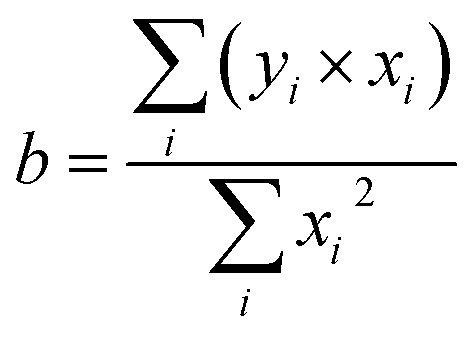
where *b* is the linear regression coefficient; *y*_*i*_ – the *i*-th value of the dependent variable *y*; *x*_*i*_ is the *i*-th value of the independent variable *x*.

The calculated by means of the CV-assisted algorithm *K*_SV_ value for the chemosensor 2 was equal to 583 340 M^−1^, which correlates well with the data obtained by the spectrofluorometer-assisted method (*K*_SV_ = 467 274 M^−1^). While the anthracene-based chemosensor 1 exhibited an almost 5 times higher constant calculated by using CV-assisted algorithm when compared to spectrofluorometer-assisted measurements (*K*_SV_ = 68 050 M^−1^*vs. K*_SV_ = 14 028 M^−1^). In addition, for the sensor 2 the limit of detection (LOD) was calculated by using CV-assisted algorithm (LOD = 436 μg L^−1^, 415 ppb) and spectrofluorometer-assisted algorithm (143 μg L^−1^, 136 ppb) (see ESI†).

Next, based on the observed above exceptionally good turn-off fluorescence response of chemosensors 1 and 2 toward TNT we decided to focus on studying the molecular quenching mechanism in aqueous media in more details.

Thus, in the presence of this nitro-analyte in aqueous media a strong fluorescence quenching of the fluorescence was observed for the both chemosensors 1 and 2. The linear behavior of Stern–Volmer plots was observed, suggesting the prevalence of only one quenching mechanism. Such a mechanism should differ from the quenching mechanism in non-polar solvents because of the poor excimer quenching outcome. One of the possible explanations of the different quenching mechanisms could lie in possible micelles/micelles-like particles formation of the chemosensors 1–2 in aqueous organic media.

To examine the possible micelles/micelle-like particles formation, we utilized DLS experiments in DMSO : H_2_O [1 : 1 (v/v)] solution. In these experiments, stepwise diluted solutions of compounds 1–2 in concentrations from 1 × 10^−4^ to 5 × 10^−6^ M were used. Particles formation was observed for both compounds 1 and 2 in all the concentration ranges, but it should be noted that nanoparticles quantity decreased upon the concentration decreasing. Thus, the results obtained at the concentration of sensors less than 1 × 10^−5^ M are not reliable because of their high deviation (Fig. S6†). The observed average particle size for both chemosensors 1–2 was approximately 180 nm, and it does not depend significantly on the concentration of these sensors. Monomodal and small particle size distribution was additionally supported by the polydispersity index (PDI) equal to 0.1 (Fig. S7†). It is worthy to mention that we didn't observe micelles/micelles-like particles formation in pure DMSO and cyclohexane.

As a second step, DLS studies of particles behavior in the presence of nitro-quenchers were carried out with use of pure acetonitrile and DNT solution in acetonitrile (see ESI, Section 6†). DNT was chosen instead of TNT due to the faster decomposition of TNT in the solution. It was revealed that pure acetonitrile leads to micelles/micelle-like particles decomposition with further re-aggregation into larger particles which compromises their stability (see ESI, Fig. S8A and S9A†). On the contrary, in the presence of acetonitrile solution of DNT, particles retain stable even at high concentrations of acetonitrile (see ESI, Fig. S8B and S9B†). It can be suggested that lipophilic nucleus of DNT intrudes into particle while polarizable nitro groups remain outside providing electrostatic stabilization.

Hence, we could conclude the following: from one side, we did not observe the decomposition of micelles/micelle-like particles of compounds 1–2 by DNT, and on the other side in DMSO : H_2_O [1 : 1 (v/v)] solution these stable particles were involved in interactions with nitro-aromatic quenchers (NAQ) to result in the dramatic fluorescence quenching which as it was reported earlier for other types of micellar chemosensors.^51,52^

**Fig. 4:**
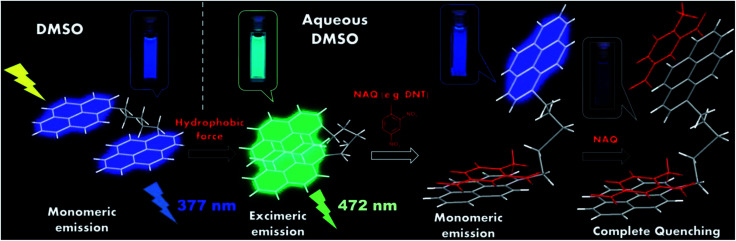
Chemosensing mechanism of *bolas*-type compounds 1 and 2. The proposed bent molecular geometry of the excimer and fluorescence quenching mechanism of the *bolas*-like compounds in aqueous organic media.

The features of the excimer and its quenching by NAE in a nonpolar medium were described above. In the aqueous environment, the Van der Waals' interactions between “hostile” dipoles of the solvent force the non-polar molecules of 1–2 to bend over the alkane linker in order to keep some non-polar “friendly” environment inside the sensor cavity. In this conformation, the bulky PAH ends of the molecules approach each other, forming a bent-shape molecular geometry (Fig. 4), which was stabilized by the induced dipole moment. In this outcome, the excimer formation is favorable and should dominate in the emission spectrum of compounds 1 and 2 in aqueous organic solvents such as DMSO : H_2_O [1 : 1 (v/v)], which was observed in our experiments (Fig. 2).

Thus, a dramatic quenching of the monomer fluorescence should be observed because of the formation of “NAE-PAH” donor–acceptor complex and the simultaneous destruction of the excimer structure and the quenching of the excimer emission (Fig. 4). Moreover, because of the ability of some NAE to form “1 : 2” complexes with PAHs,^22^ the PAH-based sensors 1–2 will act as molecular *bolas via* chelating/“catching” the nitro-aromatic quenchers (NAQ) to from 1 : 2 “NAQ : PAH” chelate complex. As a result of the formation of such complexes, a complete excimer fluorescence quenching will be observed (Fig. 4). Indeed, in our experiments in aqueous conditions in the case of compound 2 we observed a very pronounced excimer fluorescence quenching with a high value of Stern–Volmer quenching constants. Altogether, these observations revealed the *bolas*-type analyte–sensor interaction mechanisms in aqueous solvents, which nevertheless require further detailed investigation.

## Conclusions

In summary, we described tunable *bolas*-type PAH-based chemosensors which provide a simple, fast, and convenient way for the detection of common nitroaromatic explosives (2,4-DNT and 2,4,6-TNT) in aqueous solutions. The remarkable photochemical properties of these *bolas*-type chemosensors allowed for the creation of a computer vision-assisted algorithm for the “turn-off” fluorescence detection of nitro-explosives. A conventional digital camera and a conventional 365 nm UV lamp as well as an ordinary PC/laptop or smartphone, are all that is needed for the developed method. Although the detailed photochemistry of the *bolas*-type chemosensors requires further investigation, we have shown that their outstanding excimer quenching by TNT and DNT can be used in CV algorithm processing with greater sensitivity (*K*_SV_ value) than spectrometer-assisted measurements. We hope our report represents the start of an upward trend in imaging technology along with enhanced machine-based decision-making principles, as manufacturers and academia seek to exploit new aspects of the physics of the explosive detection process. Coupling effective fluorescent chemosensors and the advances in computer vision is aiming towards smarter decision-making detection systems that will complement and re-enforce the role of the human operator. While the protocols described in this report were optimized for nitro-explosives detection, with a rational chemical modification, the introduced method is considered to be similarly applicable for the other analyte analysis, for instance, in food quality analysis, ecological monitoring or medicinal and pharmaceutical applications.

## Conflicts of interest

There are no conflicts to declare.

## Supplementary Material

RA-011-D1RA03108B-s001

RA-011-D1RA03108B-s002
