# Supplementary material for: Computer vision vs. spectrofluorometer-assisted detection of common nitro-explosive components with bola-type PAH-based chemosensors
Source: RSC Adv. 2021 Jul 27;11(42):25850–7. doi: 10.1039/d1ra03108b (PMC9037216; doi:10.1039/d1ra03108b)
Supplement: RA-011-D1RA03108B-s001 [file RA-011-D1RA03108B-s001.pdf]

Electronic Supplementary Material (ESI) for RSC Advances.
This journal is © The Royal Society of Chemistry 2021

# Electronic Supplementary Information

## Computer vision *vs.* spectrofluorometer-assisted detection of common nitro-explosive components with *bola*-type PAH-based chemosensors

INDEX

**Experimental Procedures** ........ **S1**
**Supplementary Figures** ........ **S4**
**Supplementary Tables** ........ **S9**
**Image processing algorithm (computer vision) in the Mathcad programming** ........ **S11**

## EXPERIMENTAL PROCEDURES:

### *1. Materials and equipment*

All reagents and solvents were purchased from commercial sources and used without additional purification. DMSO and acetonitrile solvents were HPLC-grade. Deionized water was obtained using a Millipore Simplicity UV Water Purification System (Merck Millipore, France).

### *2.Synthesis of bolas-type molecules.*

**1,5-bis(9-anthryl)pentane.** The mixture of anthracene-9-carbaldehyde (0.6 g, 2.91 mmol) and acetone (0.11 ml, 1.45 mmol) was dissolved in 20 ml of ethanol and added to the solution of KOH (1.22 g, 21.82 mmol) in 20 ml of ethanol. The reaction mixture was stirred at room temperature for one hour. The formed precipitate was filtered, washed with water, and recrystallized from benzene-chloroform. The product was yellow crystals of 1,5-bis (9-anthryl)-l,4-pentadien-3-one. Next, it was mixed with zinc dust (4.54 g, 69.39 mmol) in 30 ml of acetic acid and refluxed under stirring for 2 hours in order to reduce* the ethylenic part of this ketone. The reaction mixture was poured into water and extracted with dichloromethane. The extract was dried over $Na_2SO_4$, evaporated under a vacuum, and recrystallized from carbon tetrachloride. The formed ketone (0.4 g, 0.91 mmol) was reduced by refluxing for two hours in triethylene glycol (20 ml) containing hydrazine (0.3 ml, 7.75 mmol) and KOH (0.19 g, 3.42 mmol). The reaction mixture was heated without the condenser at 195 °C for two hours in order to remove the excess amounts of hydrazine and water. Next, the mixture was cooled, added to water, and extracted with benzene. The extract was dried over $Na_2SO_4$ and evaporated under a vacuum. The product was purified by flash chromatography on a silica gel column (Alfa Aesar Silica Gel 60) with dichloromethane-hexane (1:4) as eluent. The eluate was evaporated under a vacuum, and then the residue was recrystallized from ethanol. The collected yellow crystals of 1,5-Bis(9-anthryl)-pentane were stored in the dark at room temperature. Yield 300 mg (0.71 mmol, 49%); mp: 173-175 °C; $^1$H NMR (400 MHz, $CDCl_3$): δ 1.88 – 1.98 (m, 6 H), 3.65 – 3.68 (m, 4 H), 7.47 – 7.54 (m, 8 H), 8.02 – 8.04 (m, 4 H), 8.28 – 8.31 (m, 4 H), 8.36 (s, 2 H).

**1,5-bis(pyren-1-yl)pentane.** The mixture of 1-pyrenecarbaldehyde (0.3 g, 1.3 mmol) and acetone (0.05 ml, 0.65 mmol) was dissolved in 20 ml of n-butanol and added to the solution of KOH (0.55 g, 9.77 mmol) in 20 ml of ethanol. The reaction mixture was stirred at 50 °C for 12 hours. The formed precipitate was filtered, washed with water, and recrystallized from benzene-chloroform. The product was red crystals of 1,5-bis(pyren-1-yl)-l,4-pentadien-3-one. Next, it was mixed with zinc dust (1.89 g, 28.88 mmol) in 30 ml of acetic acid and refluxed under stirring for two hours in order to reduce* the ethylenic part of this ketone. The reaction mixture was poured into water and extracted with dichloromethane. The extract was dried over $Na_2SO_4$, evaporated under a vacuum, and recrystallized from carbon tetrachloride. The formed ketone (0.19 g, 0.38 mmol) was reduced by refluxing for two hours in triethylene glycol (20 ml) containing hydrazine (0.13 ml, 3.23 mmol) and KOH (0.08 g, 1.43 mmol). The reaction mixture was heated without the condenser at 195 °C for two hours in order to remove the excess amounts of hydrazine and water. Next, the mixture was cooled, added to water, and extracted with benzene. The extract was dried over $Na_2SO_4$ and evaporated under a vacuum. The product was purified by flash chromatography on a silica

* Alternatively, reduction can be done in a hydrogen atmosphere (3 atm, rt) on a Pd(10%)/C catalyst

gel column (Alfa Aesar Silica Gel 60) with dichloromethane-hexane (1:4) as eluent. The eluate was evaporated under a vacuum, and then the residue was recrystallized from ethanol. The collected dark orange crystals of 1,5-Bis(pyren-1-yl)-pentane were stored in the dark at room temperature. Yield 160 mg (0.34 mmol, 52%); mp: 188-189 °C; $^{1}H$ NMR (400 MHz, $CDCl_3$): δ 1.62 - 1.70 (m, 2 H), 1.91 - 1.99 (m, 4 H), 3.32 - 3.36 (m, 4 H), 7.82 (d, *J*=7.78 Hz, 2 H), 7.96 - 8.01 (m, 6 H), 8.05 - 8.07 (m, 4 H), 8.12 - 8.15 (m, 4 H), 8.25 (d, *J*=9.29 Hz, 2 H).

***3. Absorption and emission spectroscopies***

Emission and excitation spectra were measured on the Horiba FluoroMax-4 spectrofluorometer. UV-Vis absorption spectra were recorded on the Shimadzu UV-1600 spectrophotometer.

***4. Quenching experiments (spectrofluorometer setup)***

Fluorescence titration experiments were carried out on the Horiba-Fluoromax-4 spectrofluorometer. All titration experiments were conducted in a standard 1 cm, 3 ml quartz cuvettes (Hellma Optik GmbH Jena, Germany). The fluorescence titration experiment started with the preparation of fluorophore solutions by dissolving sensors **1** or **2** compounds in DMSO:$H_2O$ [1:1 (v/v)]. The fluorescence quenching was measured by Single Point method with $\lambda_{Ex}$ = 353 nm, $\lambda_{Em}$ = 507 nm for sensor **1** and $\lambda_{Ex}$ = 353 nm, $\lambda_{Em}$ = 472 nm for sensor **2**. 3 ml of the sensor solutions ($1\times10^{-5}$ M for sensor **1** and $1\times10^{-6}$ M for sensor **2**) were placed in quartz cells following by stepwise addition of 10-15 aliquots of the analyte (2,4,6-Trinitrotoluene (TNT)) dissolved in acetonitrile with concentration $1\times10^{-3}$ M for sensor **1** and $1\times10^{-4}$ M for sensor **2**.

Analysis of fluorescence emission intensity was performed on the base of a Stern-Volmer equation (1):

$$\frac{I_0}{I} = 1 + K_{SV} \times [Q] \quad (1)$$

where $I_0$ is the intensity, or rate of fluorescence, without a quencher, I is the intensity, or rate of fluorescence, with a quencher, $K_{SV}$ is the Stern-Volmer quenching constant for complex formation, and [Q] is the concentration of the quencher. The quenching constant ($K_{SV}$) was calculated as the slope of the graph intensity (($I_0$/I)-1) versus the concentration of the quencher ([Q]).

***5. Computer vision-assisted quenching experiments (spectrofluorometer-free setup)***

The Canon D3000 Kit camera was used for the images. The fluorescence quenching experiments were carried out in 10 ml glass vials (Medsteklo, Russia). The fluorescence quenching experiment started with the preparation of fluorophore solutions. For sensors **1** and **2** were used DMSO:$H_2O$ [1:1 (v/v)] solutions. 6 ml of the sensor solution ($1\times10^{-5}$ M for sensor **1** and $1\times10^{-6}$ M for sensor **2)** was placed in a glass vial following by the addition of 11 aliquots of TNT in acetonitrile ($2\times10^{-3}$ for sensor **1** and $2\times10^{-4}$ M for sensor **2**). The first aliquot volume was 3μl; the second was 7μl, and the following nine aliquots were 10μl each. Imaging of both vials at once has been done in a dark chamber under UV lamp (λ=365 nm) before and after the addition of each aliquot. The images are shown in **Figure S3**.

Analysis of the fluorescence emission intensity was done by the image processing algorithm in Mathcad 15.0 packets (M020 [MC15_M020_20121127]) © Parametric Technology Corporation. The received values were plotted as the Stern-Volmer graph in a similar way described in the quenching experiments procedure. The detailed image-processing algorithm (computer vision) is shown on page S10 as the total listing of the Mathcad programming:

***6. Dynamic Light Scattering experiments***

Measurements were made on the Malvern Zetasizer Nano ZS (Malvern Panalytical, U.K.) equipped with a 4 mW He–Ne laser operating at a wavelength of 633 nm. The scattered light is detected at an angle of 173°, an optical arrangement known as non-invasive back scatter (NIBS) optic arrangement. Measurements were carried out in a quartz cell (volume is 1 ml) with round aperture at 25 °C. Data on viscosity and refractive index for solvent mixtures were taken from the literature[1]. The measurements were carried out in an automatic mode; the autocorrelation function was analyzed to assess the measurement quality.

The dynamic light scattering (DLS) experiments were carried out in different solvents: DMSO, DMSO:$H_2O$ [1:1 (v/v)] and cyclohexane. For measurements fresh solutions prepared within 1-2 hours were used. No particles were detected in pure DMSO and cyclohexane. Only fresh solutions of sensors **1–2** in aqueous DMSO showed presence of particles. However, in solutions of sensors **1–2** in a mixture of solvents, measured 24 hours after preparation, there were no particles, which may be due to their sedimentation.

To study the interaction of chemosensors **1–2** with DNT, we used their solutions in a DMSO:$H_2O$ [1:1 (v/v)] at $5\times10^{-5}$ M concentration. To this solution of sensor a solution of DNT in acetonitrile (the concentration of DNT was $1\times10^{-2}$ M) or pure acetonitrile in an appropriate amount was added.

***7. Calculation of the Limit of Detection (LOD)***

The detection limit was determined based on the data of experiments on fluorescent quenching according to the method published previously[2]. A calibration curve was plotted between the fluorescence intensity and the analyte concentration to obtain a regression curve equation (see **Fig. S4**). The limit of detection was calculated using the equation (2):

$$LOD = 3\sigma \div k \quad (2)$$

where σ is the standard deviation of the fluorophore intensity in the absence of an analyte obtained via STEYX function in Excel and k is the slope of the calibration curve.

**Sensor 1**

**Spectrofluorometer-free titration**

**LOD** = 3*1156.201977/1.79336E6 = 1.93414E-05= 19.34 µM = 4390.18 µg/L = **4.39 mg/L**

**m (1L solution DMSO:$H_2O$ (1:1))** = 500 ml × $\rho_{DMSO}$ + 500 ml × $\rho_{H2O}$ = 500 ml × 1.1 g/ml + 500 ml × 1.0 g/ml = 1050 g = 1.05 kg

4390.18 µg/L = $\frac{4390.18 \mu g}{1.05 kg}$ **= 4181.124 ppb**

**Spectrofluorometer titration**

**LOD** = 3*32656.61713/7.77747E9 = 1.25966E-05= 12.60 µM = 2859.433 µg/L = **2.86 mg/L**

2859.433 µg/L =$\frac{2859.433 \mu g}{1.05 kg}$ **= 2723.27 ppb**

**Sensor 2**

Spectrofluorometer-free CV titration

**LOD** = 3*1144.224809/1.78815E7 = 1.91968E-06 = 1.920 µM = 435.77 µg/L = **0.436 mg/L**

435.77 µg/L = $\frac{435.77 \mu g}{1.05 kg}$ **= 415.02 ppb**

**Spectrofluorometer titration**

**LOD** = 3*28724.70727/1.36637E11 = 6.30679E-07 = 0.631 µM = 143.164 µg/L = **0.143 mg/L**

143.164 µg/L = $\frac{143.164 \mu g}{1.05 kg}$ **= 136.347 ppb**

**SUPPLEMENTARY FIGURES:**

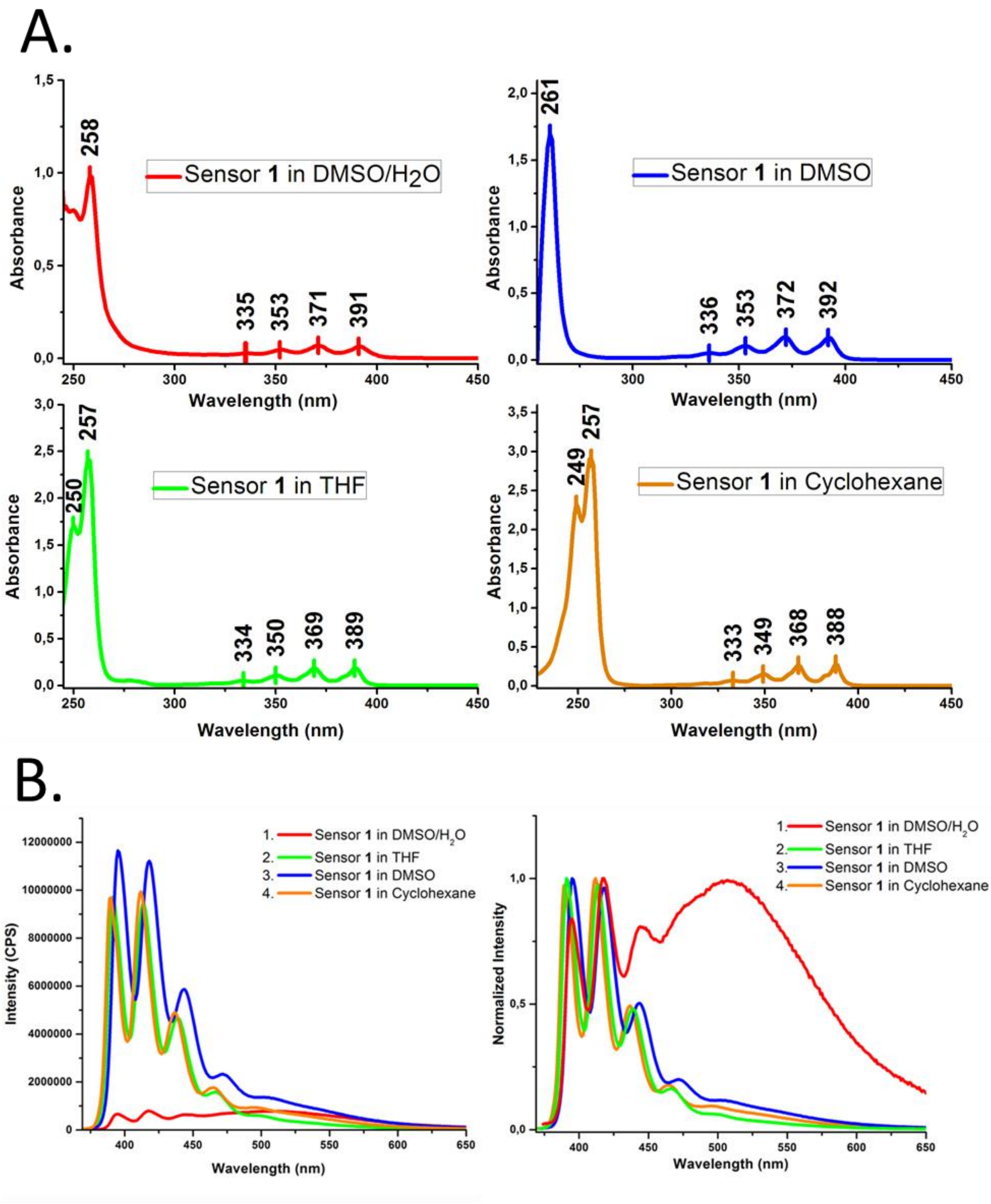


**Fig.S1** Absorption and emission spectra of sensor **1** **(**1,5-di (anthracene-9-yl) pentane**)** in different solvents **(A)** Absorption spectra of sensor **1** ($1\times10^{-5}$ M) (**B**) Emission spectra (left) and normalized emission spectra (right) of sensor **1** ($1\times10^{-5}$ M).

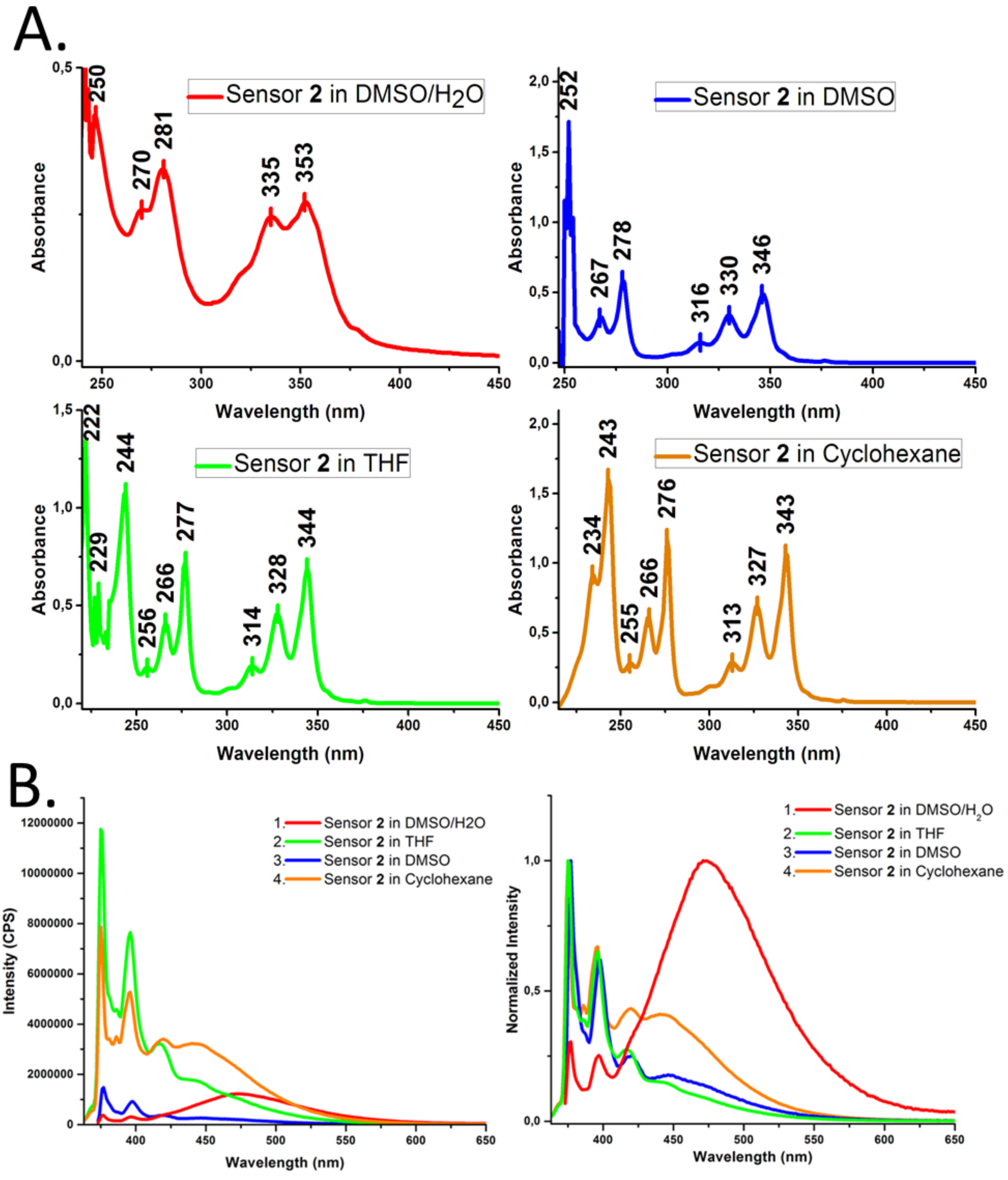


**Fig.S2** Absorption and emission spectra of sensor **2** **(**1,5-di (anthracene-9-yl) pentane**)** in different solvents **(A)** Absorption spectra of sensor **2** ($1\times10^{-6}$ M); **(B)** Emission spectra (left) and normalized emission spectra (right) of sensor **2** ($1\times10^{-6}$ M).

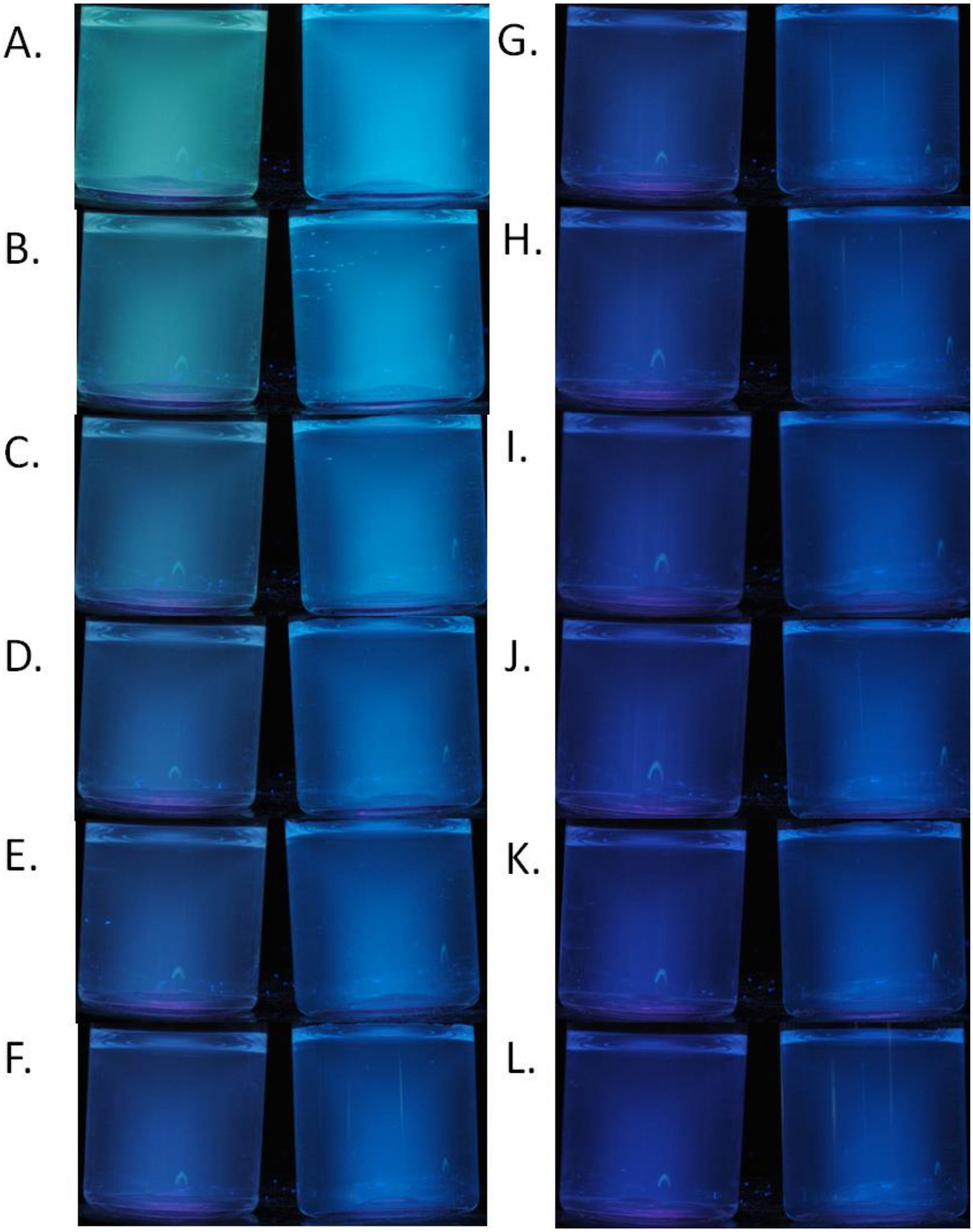


**Fig.S3** Solutions of sensor **1** ($1\times10^{-5}$ M of 1,5-di (anthracene-9-yl) pentane in the left vial) and sensor **2** ($1\times10^{-6}$ M of 1,5-di (anthracene-9-yl) pentane in the right vial) in DMSO:$H_2O$ [1:1 (v/v)] without (**A**) and with (**B-L**) stepwise added nitro explosive TNT in acetonitrile solution ($2\times10^{-3}$ M for sensor **1** and $2\times10^{-4}$ M for sensor **2**). The cumulative volume of the added TNT solution is 3 µl (B), 10 µl (C), 20 µl (D), 30 µl (E), 40 µl (F), 50 µl (G), 60 µl (H), 70 µl (I), 80 µl (J), 90 µl (K), 100 µl (L).

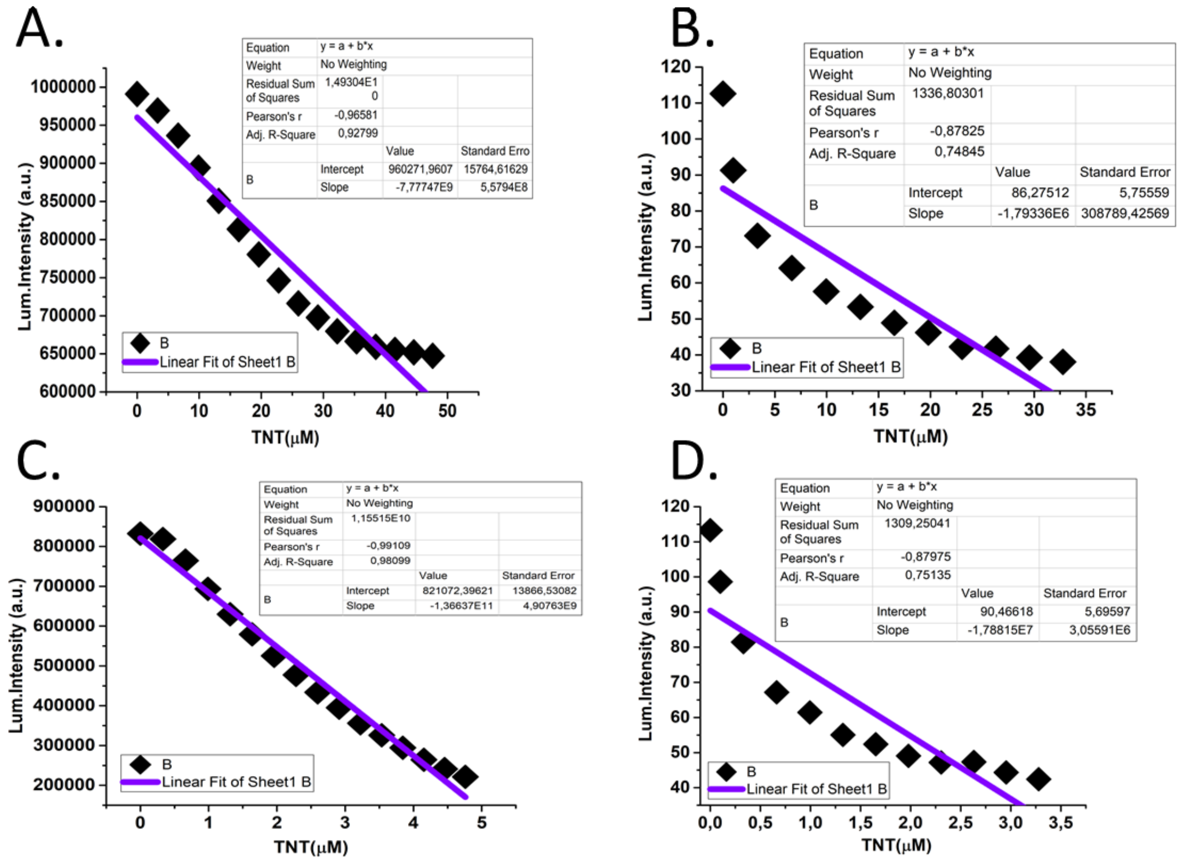


**Fig.S4** Calculation of the Limit of Detection Graph of fluorescent intensity of the sensor **1 (**1,5-di (anthracene-9-yl) pentane**)** and sensor **2 (**1,5-di (anthracene-9-yl) pentane**)**: **(A)** Fluorescent intensity of sensor **1** versus trinitrotoluene concentration determined by spectrofluorometer. **(B)** Fluorescent intensity of sensor **1** versus trinitrotoluene concentration determined via the computer vision-assisted algorithm. **(C)** Fluorescent intensity of sensor **2** versus trinitrotoluene concentration determined by spectrofluorometer **(D)** Fluorescent intensity of sensor **2** versus trinitrotoluene concentration determined via computer vision-assisted algorithm.

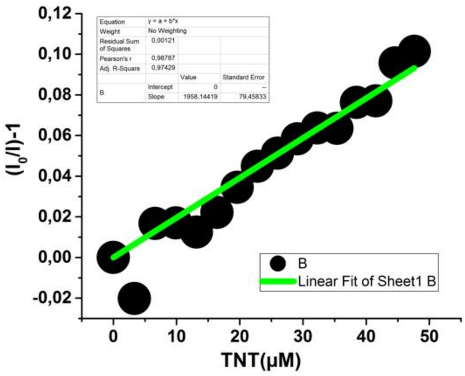


**Fig.S5** Stern-Volmer plot of emission quenching for sensor **2** in Cyclohexane

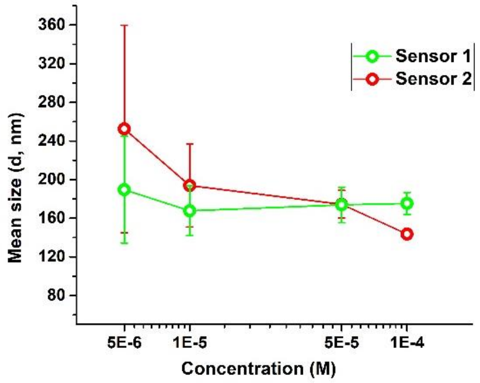


**Fig. S6.** Weighted average particle diameter plotted against concentration. The confidence intervals show the particle distribution as converted from the PDI.

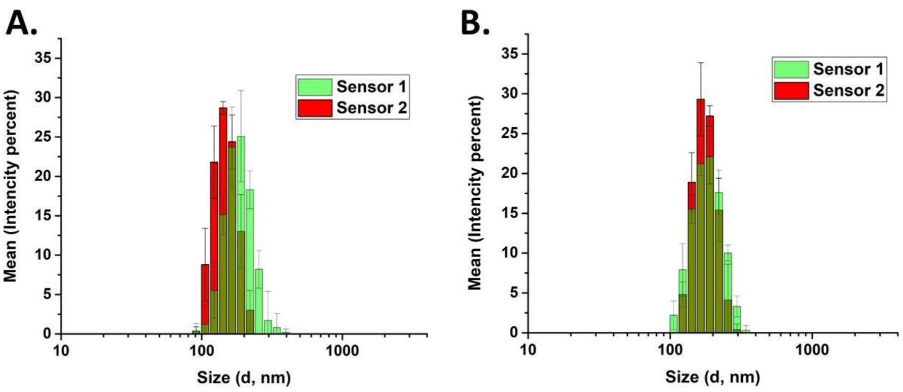


**Fig. S7.** Histogram of particle size distribution for the chemosensors **1-2** at concentrations $1\times10^{-4}$ M and $5\times10^{-6}$ M (Figures A and B, respectively).

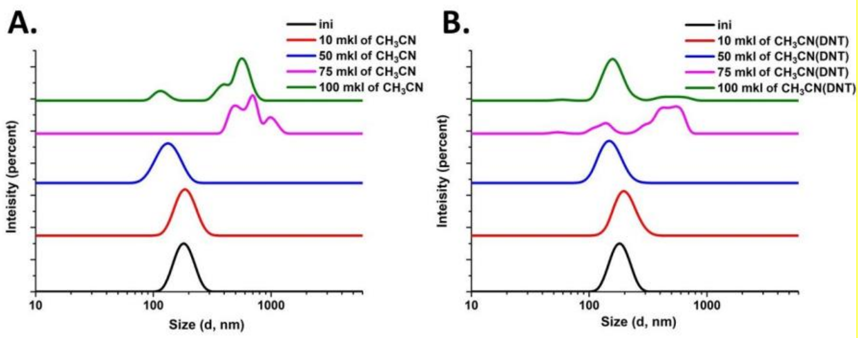


**Fig. S8.** Distribution of particles by size (recalculated by intensity) for sensor **2**, with the addition of pure acetonitrile (A) and a solution of DNT in acetonitrile (B).

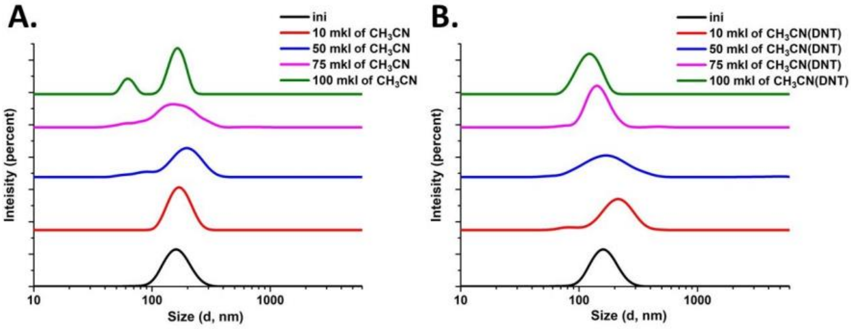


**Fig. S9.** Distribution of particles by size (recalculated by intensity) for sensor **1**, with the addition of pure acetonitrile (A) and a solution of DNT in acetonitrile (B).

**SUPPLEMENTARY TABLES:**

**Table S2** Summary of Stern-Volmer quenching constants of sensors **1-2**

| Sensor | $K_{SV}^{TNT}$ | |
|---|---|---|
| | DMSO:$H_2O$ [1:1 (v/v)] | Cyclohexane |
| **1** | $1.28\times10^{4}$ | – |
| **2** | $4.67\times10^{5}$ | $1.96\times10^{3}$ |

**Table S3** Quenching efficiency (%) of sensors **1-2**

| Sensor | TNT | |
|---|---|---|
| | DMSO:$H_2O$ [1:1 (v/v)] | Cyclohexane |
| **1** | 31.41 | – |
| **2** | 73.38 | 9.21 |

**Table S4.** Fluorescence quenching experiment of **1** upon addition TNT

| Solution of sensor **1** (solvent) | Concentration of sensor 1, M | Solution of quencher (solvent) | Concentration of quencher, M |
|---|---|---|---|
| DMSO:$H_2O$ [1:1 (v/v)] | $1.0\times10^{-5}$ M | $CH_3CN$ | $1.0\times10^{-3}$ M |

**Table S5**. Summary of the data of the fluorescence quenching experiments for the sensor **1** by using Single Point method

| **µM** | **TNT** | |
|---|---|---|
| | **($I_0$/I)-1** | **Q.Ef.,%** |
| 0.0000 | 0.00000 | 0.00 |
| 3.3223 | 0.02215 | 2.17 |
| 6.6225 | 0.05830 | 5.51 |
| 9.9010 | 0.10804 | 5.51 |
| 13.1579 | 0.16467 | 9.75 |
| 16.3934 | 0.21772 | 14.14 |
| 19.6078 | 0.26968 | 17.88 |
| 22.8013 | 0.32767 | 21.24 |
| 25.9740 | 0.38337 | 24.68 |
| 29.1262 | 0.42008 | 27.71 |
| 32.2581 | 0.45802 | 31.41 |

**Table S6.** Fluorescence quenching experiment of **1** upon addition TNT

| Solution of sensor **2** (solvent) | Concentration of sensor **2**, M | Solution of quencher (solvent) | Concentration of quencher, M |
|---|---|---|---|
| DMSO:$H_2O$ [1:1 (v/v)] | $1.0\times10^{-6}$ M | $CH_3CN$ | $1.0\times10^{-4}$ M |

**Table S7** Fluorescence quenching experiment of **2** upon addition TNT

| Solution of sensor **2** (solvent) | Concentration of sensor **2**, M | Solution of quencher (solvent) | Concentration of quencher, M |
|---|---|---|---|
| Cyclohexane | $1.0\times10^{-5}$ M | THF | $1.0\times10^{-3}$ M |

**Table S8.** Summary of the data of the fluorescence quenching experiments for sensor **2** by using Single Point method

| **µM** | **TNT (DMSO:$H_2O$ [1:1 (v/v)])** | | **µM** | **TNT (Cyclohexane)** | |
|---|---|---|---|---|---|
| | **($I_0$/I)-1** | **Q.Ef.,%** | | **($I_0$/I)-1** | **Q.Ef.,%** |
| 0.0000 | 0.00000 | 0.00 | 0.0000 | 0 | 0.00 |
| 0.3322 | 0.01665 | 1.64 | 3.3223 | -0.02015 | -2.06 |
| 0.6623 | 0.08900 | 8.17 | 6.6225 | 0.01675 | 1.65 |
| 0.9901 | 0.19999 | 8.17 | 9.9010 | 0.01699 | 1.65 |
| 1.3158 | 0.32177 | 16.67 | 13.1579 | 0.01255 | 1.67 |
| 1.6393 | 0.43680 | 24.34 | 16.3934 | 0.02218 | 1.24 |
| 1.9608 | 0.58379 | 30.40 | 19.6078 | 0.03454 | 2.17 |
| 2.2801 | 0.74259 | 36.86 | 22.8013 | 0.04488 | 3.34 |
| 2.5974 | 0.91991 | 42.61 | 25.9740 | 0.05129 | 4.30 |
| 2.9126 | 1.10653 | 47.91 | 29.1262 | 0.05841 | 4.88 |
| 3.2258 | 1.33691 | 57.21 | 32.2581 | 0.06374 | 5.99 |
| 3.5370 | 1.55404 | 60.85 | 35.3698 | 0.06358 | 5.98 |
| 3.8462 | 1.82535 | 64.61 | 38.4615 | 0.07628 | 7.09 |
| 4.1534 | 2.15062 | 68.26 | 41.5335 | 0.07709 | 7.16 |
| 4.4586 | 2.45576 | 71.06 | 44.5860 | 0.09573 | 8.74 |
| 4.7619 | 2.75607 | 73.38 | 47.6190 | 0.1014 | 9.21 |

**IMAGE PROCESSING ALGORITHM**

**Image-processing algoritm (computer vision) is presented as the total listing of the Mathcad programming:**

make a vector of values to read from files

$$V := \begin{pmatrix} \text{"IMG0.bmp"} \\ \text{"IMG1.bmp"} \\ \text{"IMG2.bmp"} \\ \text{"IMG3.bmp"} \\ \text{"IMG4.bmp"} \\ \text{"IMG5.bmp"} \\ \text{"IMG6.bmp"} \\ \text{"IMG7.bmp"} \\ \text{"IMG8.bmp"} \\ \text{"IMG9.bmp"} \\ \text{"IMG10.bmp"} \\ \text{"IMG11.bmp"} \end{pmatrix}$$

read cyclically the images in the form of the matrix BEF tensor

```
BEF := | for i ∈ 0..11
       |    A_i ← READRGB(V_i)
       | A
```

determine the number of columns in the composite matrix of RGB colors (BEF)

$a := cols(BEF_0) = 2472$

$\frac{a}{3} = 824$

```
M := | for i ∈ 0..11
     |   | for j ∈ 0..rows(BEF_i) − 1
     |   |    for k ∈ 0..cols(BEF_i)/3 − 1
     |   |       A_{j,k} ← (BEF_i)_{j,k}·0.2126 + (BEF_i)_{j,k+824}·0.7152 + (BEF_i)_{j,k+824·2}·0.0722
     |   | M_i ← A
     | M
```

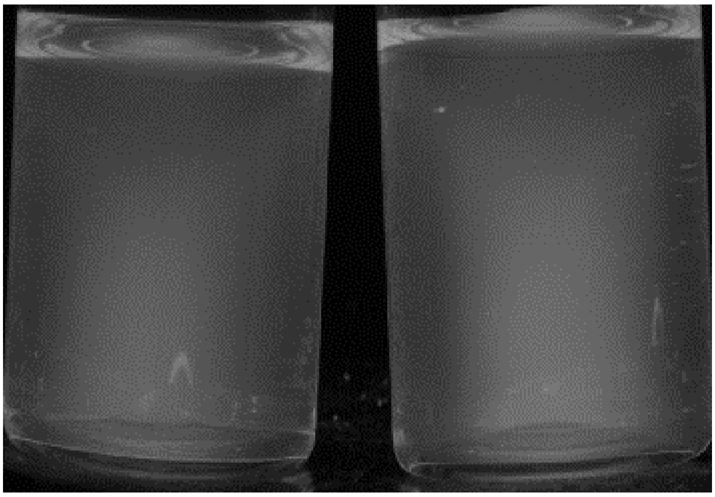

$M_2$

manually select submatrices using the image of the matrix $M_2$

$FP0 := \text{submatrix}(M_2, 55, 348, 40, 331)$

$FP := \text{submatrix}(M_2, 49, 342, 459, 750)$

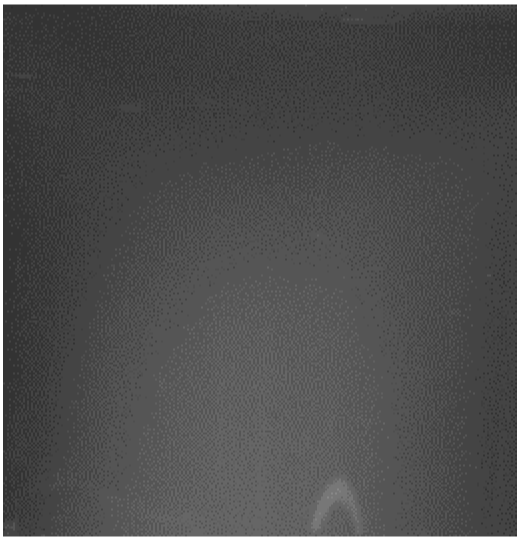

FP0

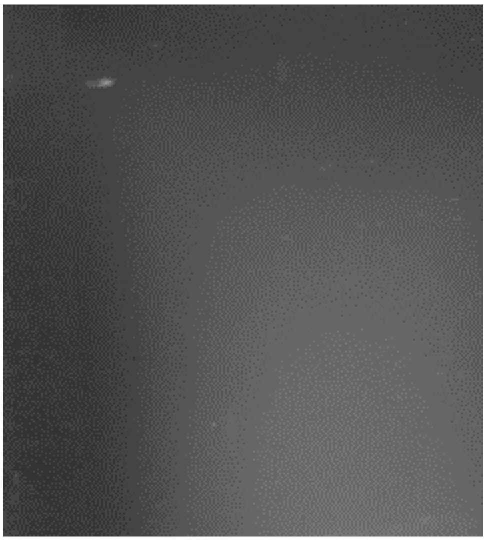

FP

According to the manually obtained coordinates, select 12 submatrices from the images of each vial

$$ANT := \left| \begin{array}{l} \text{for } i \in 0..11 \\ \quad M_i \leftarrow \text{submatrix}\left(M_{\cdot,1}, 55, 348, 40, 331\right) \\ M \end{array} \right.$$

$$PYR := \left| \begin{array}{l} \text{for } i \in 0..11 \\ \quad M_i \leftarrow \text{submatrix}\left(M_{\cdot,1}, 49, 342, 459, 750\right) \\ M \end{array} \right.$$

calculate the vector of the average values of the tensor of the matrices ANT and PYR

$$Y_{ANT} := \left| \begin{array}{l} \text{for } b \in 0..11 \\ \quad \left| \begin{array}{l} a_b \leftarrow 0 \\ \text{for } i \in 0..\text{rows}\left(ANT_b\right) - 1 \\ \quad \text{for } j \in 0..\text{cols}\left(ANT_b\right) - 1 \\ \quad\quad a_b \leftarrow a_b + \left(ANT_b\right)_{i,j} \\ a_b \leftarrow \dfrac{a_b}{\text{rows}\left(ANT_b\right) \cdot \text{cols}\left(ANT_b\right)} \end{array} \right. \\ a \end{array} \right.$$

$Y_{ANT} =$

| | 0 |
|---|---|
| 0 | 112.577 |
| 1 | 91.294 |
| 2 | 73.111 |
| 3 | 64.139 |
| 4 | 57.623 |
| 5 | 53.362 |
| 6 | 48.927 |
| 7 | 46.199 |
| 8 | 42.263 |
| 9 | 41.735 |
| 10 | 39.227 |
| 11 | 38.055 |

$$Y_{PYR} := \left| \begin{array}{l} \text{for } b \in 0..11 \\ \quad \left| \begin{array}{l} a_b \leftarrow 0 \\ \text{for } i \in 0..\text{rows}\left(PYR_b\right) - 1 \\ \quad \text{for } j \in 0..\text{cols}\left(PYR_b\right) - 1 \\ \quad\quad a_b \leftarrow a_b + \left(PYR_b\right)_{i,j} \\ a_b \leftarrow \dfrac{a_b}{\text{rows}\left(PYR_b\right) \cdot \text{cols}\left(PYR_b\right)} \end{array} \right. \\ a \end{array} \right.$$

$Y_{PYR} =$

| | 0 |
|---|---|
| 0 | 113.285 |
| 1 | 98.605 |
| 2 | 81.465 |
| 3 | 67.178 |
| 4 | 61.416 |
| 5 | 55.053 |
| 6 | 52.361 |
| 7 | 49.067 |
| 8 | 47.199 |
| 9 | 47.334 |
| 10 | 44.347 |
| 11 | 42.445 |

vectors Y are converted to the form (Y0 / Yi)-1 in accordance with the Stern-Volmer equation

$$Y_{ANTsv} := \left| \begin{array}{l} \text{for } i \in 0..11 \\ \quad a_i \leftarrow \dfrac{Y_{ANT_0}}{Y_{ANT_i}} - 1 \\ a \end{array} \right.$$

$Y_{ANTsv} =$

| | 0 |
|---|---|
| 0 | 0 |
| 1 | 0.233 |
| 2 | 0.54 |
| 3 | 0.755 |
| 4 | 0.954 |
| 5 | 1.11 |
| 6 | 1.301 |
| 7 | 1.437 |
| 8 | 1.664 |
| 9 | 1.697 |
| 10 | 1.87 |
| 11 | 1.958 |

$$Y_{PYRsv} := \left| \begin{array}{l} \text{for } i \in 0..11 \\ \quad a_i \leftarrow \dfrac{Y_{PYR_0}}{Y_{PYR_i}} - 1 \\ a \end{array} \right.$$

$Y_{PYRsv} =$

| | 0 |
|---|---|
| 0 | 0 |
| 1 | 0.149 |
| 2 | 0.391 |
| 3 | 0.686 |
| 4 | 0.845 |
| 5 | 1.058 |
| 6 | 1.164 |
| 7 | 1.309 |
| 8 | 1.4 |
| 9 | 1.393 |
| 10 | 1.554 |
| 11 | 1.669 |

create a vector containing the volume of the quencher solution

$$V := \begin{pmatrix} 0L \\ 3\cdot10^{-6}L \\ 7\cdot10^{-6}L \\ 10\cdot10^{-6}L \\ 10\cdot10^{-6}L \\ 10\cdot10^{-6}L \\ 10\cdot10^{-6}L \\ 10\cdot10^{-6}L \\ 10\cdot10^{-6}L \\ 10\cdot10^{-6}L \\ 10\cdot10^{-6}L \\ 10\cdot10^{-6}L \end{pmatrix}$$

Create X vectors of current TNT concentrations

$$X_{ANT} := \left| \begin{array}{l} \text{for } i \in 0..11 \\ \quad a_i \leftarrow \dfrac{\left(\sum_{n=0}^{i} V_n\right)\cdot 2\cdot10^{-3}\dfrac{mol}{L}}{\sum_{n=0}^{i} V_n + 6\cdot10^{-3}L} \\ a \end{array} \right.$$

$X_{ANT} =$

| | 0 |
|---|---|
| 0 | 0 |
| 1 | $9.995\cdot10^{-7}$ |
| 2 | $3.328\cdot10^{-6}$ |
| 3 | $6.645\cdot10^{-6}$ |
| 4 | $9.95\cdot10^{-6}$ |
| 5 | $1.325\cdot10^{-5}$ |
| 6 | $1.653\cdot10^{-5}$ |
| 7 | $1.98\cdot10^{-5}$ |
| 8 | $2.306\cdot10^{-5}$ |
| 9 | $2.632\cdot10^{-5}$ |
| 10 | $2.956\cdot10^{-5}$ |
| 11 | $3.279\cdot10^{-5}$ |

$\cdot\dfrac{mol}{L}$

$$X_{PYR} := \left| \begin{array}{l} \text{for } i \in 0..11 \\ \quad a_i \leftarrow \dfrac{\left(\sum_{n=0}^{i} V_n\right)\cdot 2\cdot 10^{-4}\dfrac{mol}{L}}{\sum_{n=0}^{i} V_n + 6\cdot 10^{-3} L} \\ a \end{array}\right.$$

$X_{PYR} =$ (in $\frac{mol}{L}$)

| | 0 |
|---|---|
| 0 | 0 |
| 1 | $9.995\cdot10^{-8}$ |
| 2 | $3.328\cdot10^{-7}$ |
| 3 | $6.645\cdot10^{-7}$ |
| 4 | $9.95\cdot10^{-7}$ |
| 5 | $1.325\cdot10^{-6}$ |
| 6 | $1.653\cdot10^{-6}$ |
| 7 | $1.98\cdot10^{-6}$ |
| 8 | $2.306\cdot10^{-6}$ |
| 9 | $2.632\cdot10^{-6}$ |
| 10 | $2.956\cdot10^{-6}$ |
| 11 | $3.279\cdot10^{-6}$ |

Stern-Volmer plot for the sensor 1

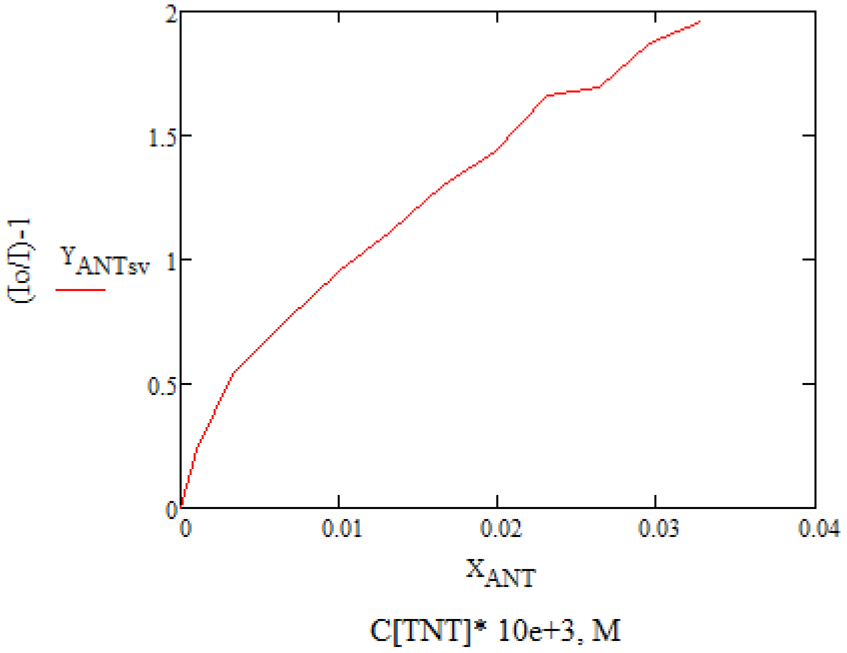

Stern-Volmer plot for the sensor 2

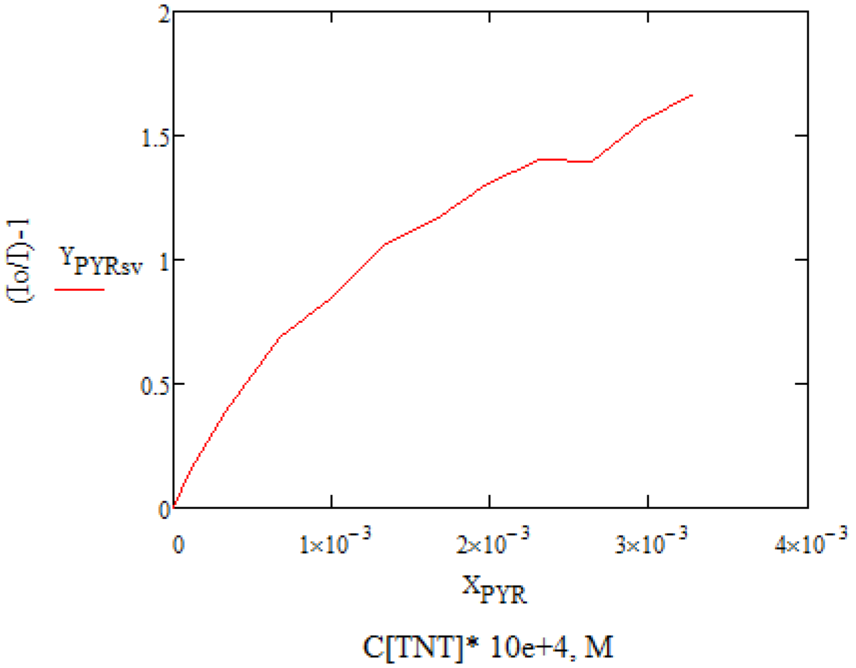


using the method of the least squares for the equation y = b * x

$i := 0..11$

$$b_{PYR} := \frac{\sum_i \left(X_{PYR_i} \cdot Y_{PYRsv_i}\right)}{\left[\sum_i \left(X_{PYR_i}\right)^2\right]} = 583339.97 \cdot \frac{L}{mol} \qquad b_{ANT} := \frac{\sum_i \left(X_{ANT_i} \cdot Y_{ANTsv_i}\right)}{\left[\sum_i \left(X_{ANT_i}\right)^2\right]} = 68049.637 \cdot \frac{L}{mol}$$

1 R. G. Lebel and D. A. I. Goring, *J. Chem. Eng. Data*, 1962, **7**, 100–101.
2 A. Shrivastava and V. Gupta, *Chronicles Young Sci.*, 2011, **2**, 21.
